# Integrating Complementary Medicine Into the Care of Childhood Cancer Survivors: A Brief Report on the Preliminary Framework and Implementation of an Educational Program

**DOI:** 10.3389/fresc.2022.897677

**Published:** 2022-06-15

**Authors:** Chun Sing Lam, Kwok Yin Au, Hing Yu Hung, Ho Wing Chou, Alex Wing Kwan Leung, Chi Kong Li, Ho Kee Koon, Yin Ting Cheung

**Affiliations:** ^1^School of Pharmacy, Faculty of Medicine, The Chinese University of Hong Kong, Hong Kong, Hong Kong SAR, China; ^2^Hong Kong Institute of Integrative Medicine, The Chinese University of Hong Kong, Hong Kong, Hong Kong SAR, China; ^3^School of Chinese Medicine, Faculty of Medicine, The Chinese University of Hong Kong, Hong Kong, Hong Kong SAR, China; ^4^Department of Paediatrics, Faculty of Medicine, The Chinese University of Hong Kong, Hong Kong, Hong Kong SAR, China; ^5^Department of Paediatrics and Adolescent Medicine, The Hong Kong Children's Hospital, Hong Kong, Hong Kong SAR, China; ^6^Hong Kong Hub of Paediatric Excellence, The Chinese University of Hong Kong, Hong Kong, Hong Kong SAR, China

**Keywords:** pediatric cancer, cancer survivorship, integrative medicine, complementary medicine, Chinese medicine

## Abstract

**Background:**

Existing educational programs typically include limited information on traditional, complementary, and integrative medicine (TCIM) for survivors of childhood cancer.

**Objectives:**

This brief report presents the preliminary results of an educational program that aims to promote the safe and effective use of Chinese medicine (CM) among survivors in Hong Kong.

**Methods:**

Survivors of childhood cancer, their caregivers, and oncology practitioners were invited to participate in a program that consists of two didactic seminars and a written educational booklet that disseminated information on the use of CM. A structured questionnaire was used to evaluate participants' receptivity toward and perceived relevance of the program. The Reach, Effectiveness, Adoption, Implementation, and Maintenance (RE-AIM) framework was used to discuss the impact of the intervention.

**Results:**

Reach: A total of 174 participants attended the seminars, and the seminar recording received over 380 views on social media platforms since April 2021. The hardcopy of the educational booklet was distributed to 43 recipients. The web-version of the booklet was sent to 67 participants and downloaded 143 times. Efficacy: The majority found that the content of the seminar useful (mean score = 5.04/6 points), especially the CM exercise (mean score = 4.88/6 points) and dietary advice (mean score = 4.99/6 points). Intention to adopt: The survivors (or their caregivers) reported that they would adopt advice on food therapies (83.3%) and traditional Chinese health exercises (55.6%) during survivorship.

**Conclusion:**

The preliminary data on patient preferences will be applied to further develop educational materials and to establish a TCIM referral network within the cancer survivor community.

## Introduction

Advances in contemporary treatment strategies have led to a significant improvement in the 5 year survival rate of children with cancer, leading to an emerging population of survivors globally (1). However, survivorship comes at the cost of developing treatment-related late effects, such as endocrine, cardiac, musculoskeletal, and neurological complications (2). The experience of chronic pain, fatigue, sleep disturbances, and other cancer-related symptoms may significantly hamper the health-related quality of life and functional outcomes of survivors (3, 4).

While some of the late effects may require lifelong pharmacological interventions (3), survivors may also turn to traditional, complementary, and integrative medicine (TCIM) to address these health issues. TCIM refers to a broad set of healthcare practices that are not part of conventional medicine and are not fully integrated into the dominant healthcare system (5). Studies have shown that cancer survivors use TCIM to manage their physical and psychological symptoms (6, 7). To promote the safe and appropriate use of TCIM, some international groups have developed recommendations to facilitate the safe and appropriate use of TCIM. For example, the International Society of Pediatric Oncology has disseminated guidelines to assimilate integrative oncology into cancer care (8). There are also reviews and training programs to educate oncology practitioners on how to provide evidence-informed TCIM treatments to cancer patients (9, 10). These published information and recommendations aim to facilitate the implementation of effective, culturally-sensitive and sustainable models of integrative care.

In Hong Kong, the use of TCIM is increasingly prevalent among patients and survivors of cancer. This popularity is fueled by the recognition of Chinese medicine practitioners as a licensed profession and the incorporation of the citywide Chinese Medicine Clinic cum Training and Research Centers into the public healthcare service in Hong Kong (11). Furthermore, the local government recently announced the establishment of the new Chinese Medicine hospital in a few years (12). However, the public may still lack knowledge and resources on how to use TCIM safely and effectively. Our team previously reported that approximately half of the Chinese survivors of childhood cancer in Hong Kong (52%) have used TCIM, with traditional Chinese medicine (CM) being the most popular modality. However, one-third of the survivors are at risk of drug-TCIM interactions during the concurrent use of TCIM and chronic medications (13). This highlights the essential need of targeted educational programs to promote the safe and effective use of TCIM modalities for survivors of childhood cancer.

Cancer survivors and their families are often highly receptive to knowledge about lifestyles optimization during the early survivorship period. Previous research has demonstrated the short-term efficacy of health education programs in increasing health-promoting behaviors, such as nutrition and physical activities, among survivors of childhood cancer (14, 15). In fact, many TCIM modalities, including traditional CM, naturopathy, and Ayurveda, also emphasize dietary habits and physical exercise as health-promotion and disease-prevention strategies (16). Despite the popularity of TCIM, existing educational programs and resources include only a limited amount of information relevant to survivors of childhood cancer.

The research-to-practice gap in translating knowledge of TCIM to practice models has been widely recognized. Health authorities, such as the National Center for Complementary and Integrative Health, support the implementation and dissemination of research on TCIM interventions (17). In recent years, there has been growing interest in the field of implementation science. For example, the Reach, Effectiveness, Adoption, Implementation and Maintenance (RE-AIM) framework conceptualizes the public health impact of an intervention as a function of five factors: reach, effectiveness, adoption, implementation, and maintenance (18). These factors are crucial in evaluating programs intended for wide-scale dissemination within a targeted population (19).

In March 2021, our group initiated a program that aimed to deliver educational resources to promote the safe and effective use of CM and to develop a framework for providing advice on CM for survivors of childhood cancer. This report aims to present: (1) an overview of the educational program; and (2) an initial evaluation of the participants' receptivity toward the program and their content preferences. We aimed to incorporate elements from the RE-AIM model to describe the impact of the program thus far, to discuss the application of these preliminary findings in advising future implementation strategies and the development of the educational program, and to identify future opportunities for integrative care in survivorship care.

## Methods

### Overview of the Educational Program

**Figure 1:**
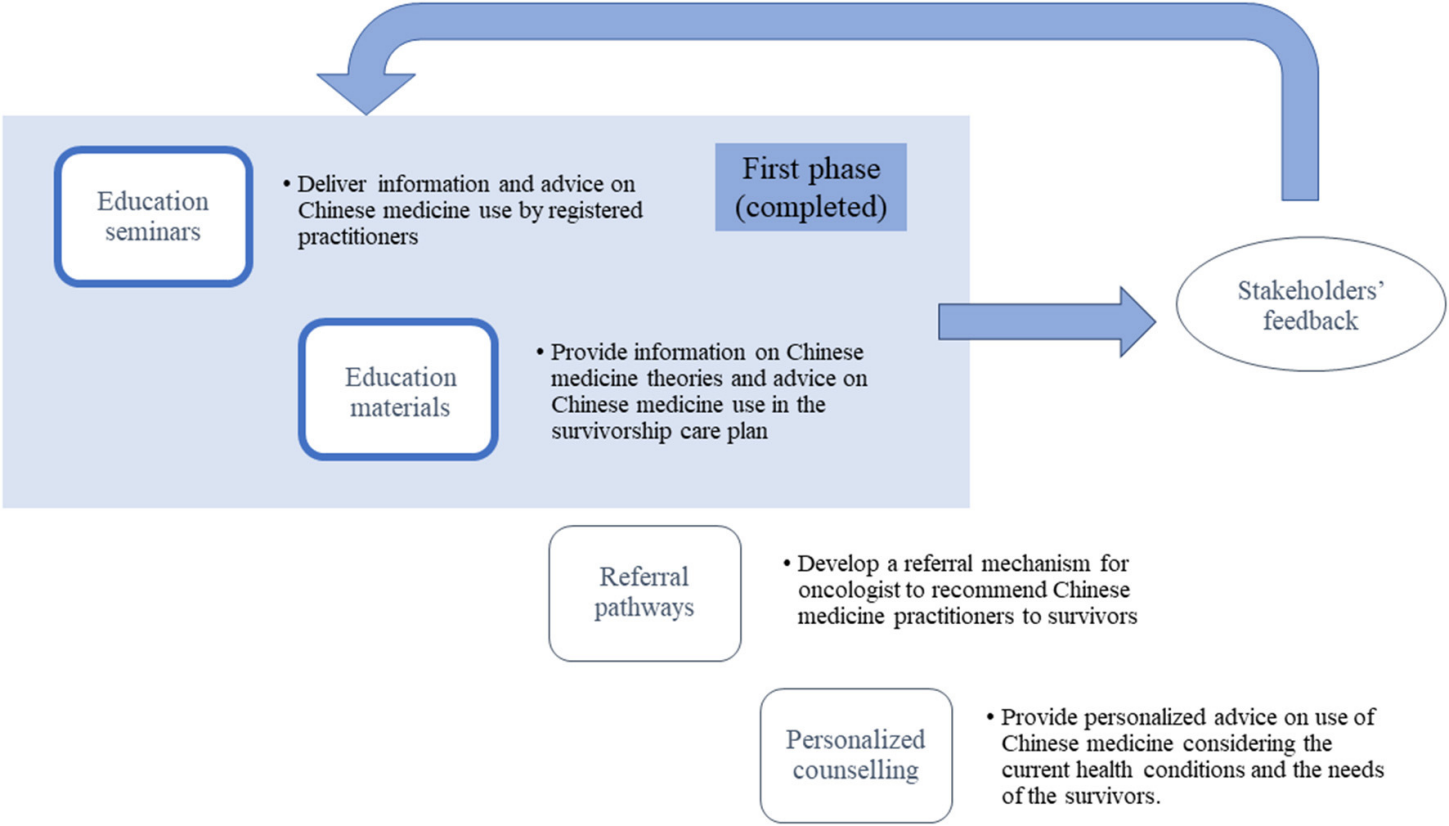
The framework of the educational program.

The educational program, entitled “Promoting Safe and Appropriate Use of TCM in Survivors of Childhood Cancer” (19B1-2/017A_R1), was supported by Hong Kong Food and Health Bureau under the Chinese Medicine Development Fund. The core study team consisted of two pediatric oncologists (AWL and CKL), two pharmacists (CSL and HWC), two licensed Chinese Medicine practitioners specializing in integrative oncology (HKK and KYA), and one methodologist (YTC). The program specifically targeted individuals who were diagnosed with cancer before the age of 18 years and had completed anti-cancer treatment (i.e., surgery, chemotherapy, radiation, and hematopoietic stem cell transplant, or targeted therapy). As the majority of patients and survivors were still under the age of 18 years, the content of the program was designed mainly for their parents and caregivers. Healthcare professionals specializing in oncology and/or TCIM were also invited to participate in the program. The overall framework of the program is presented in Figure 1.

### Educational Seminar

As part of the program, a series of didactic seminars are held to disseminate information on CM use to childhood cancer survivors and their families/caregivers. The seminars are advertised through local pediatric oncology groups, non-governmental organizations, and social media platforms (WhatsApp and Facebook). The seminars were delivered by registered CM practitioner focusing on the field of integrative oncology. The selection of seminar topics and contents was based on clinical consensus among the study team members and our previous report on the most common TCIM modalities among local cancer survivors (13).

Due to the COVID-19 pandemic, the first seminar was delivered by video conference using Zoom (Zoom Video Communications, United States) on April 15, 2021. The contents of the first seminar were mainly focused on: (1) CM perspectives on children physiology and cancer; (2) the body constitution characteristics according to CM theories; (3) CM principles of health promotion in cancer in both physical and psychological aspects; and (4) CM advice (dietary, herbal, and exercise) for survivor. The second seminar was delivered at the CUHK Medical Center, a non-profit, private teaching hospital, and livestreamed over Zoom. The second seminar focused on non-oral therapies (e.g., acupressure, acupuncture and health exercises) for survivors of childhood cancer.

**Figure 2:**
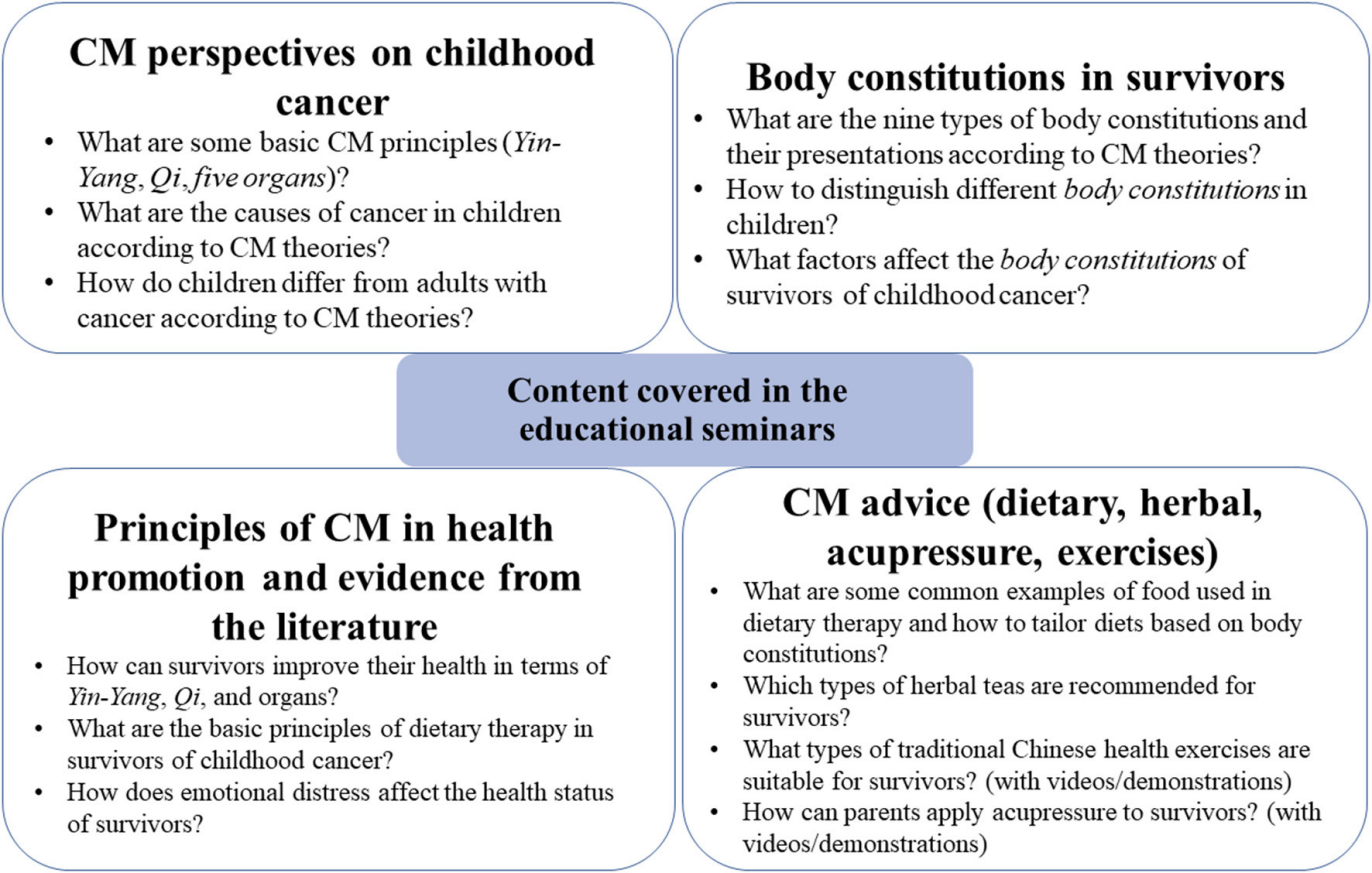
Content covered in the educational seminar.

Both seminars also involved clinical sharing of healthcare professionals and an interactive question-and-answer session at the end. Recordings of the seminars were uploaded to social media platforms and made available to the public. The presentation slides were also disseminated to the participants after the seminars. A summary of the content covered in the seminars is presented in Figure 2.

### Educational Resources as Part of the Survivorship Care Plan

A set of educational materials was developed to provide information on CM theories and advice on CM use during the post-treatment phase. The materials was integrated as a part of the survivorship care plan, which currently contains personalized information on survivors' cancer diagnosis and treatment history, as well as late effects and health promotion advice adapted from the Children Oncology Group (20, 21). The key information was focused on CM dietary therapies, herbal products, and non-oral approaches such as traditional exercises, and were developed based on clinical consensus and existing recommendations in the literature (9, 10). The materials included descriptions and illustrations of selected herbs according to the safety, suitability, cautions and contraindications for cancer survivors to use. The educational materials were distributed through local non-governmental organizations (NGOs), such as the Children Cancer Foundation and the Little Life Warriors Society, which have access to a wide network of children with cancer and play an important role as patient advocates.

### Outcomes Assessment

To evaluate attendees' receptivity toward the educational seminars, a post-event feedback questionnaire was developed using Qualtrics XM (Qualtrics, United States). The questionnaire mainly evaluated the perceived usefulness of the overall content and each topic using a seven-point scale (“0” = “not useful at all” to “6” = “extremely useful”). The participants were asked whether they were interested in joining future events and to provide any additional comments or further suggestions. We also asked individuals diagnosed with pediatric cancer, and/or their families/caregivers if they would adopt any of the advice (“yes” or “no”) provided in the seminars, including: (1) incorporating recommended foods/herbs into their diets; (2) taking herbal tea; (3) performing traditional Chinese health exercises; and (4) using acupressure/massage.

The feedback questionnaire for the written educational resources included evaluating the participants' perceived usefulness of the content and if recipients would adopt any of the advice (“yes” or “no”) included in the booklet. In addition, we also asked whether the materials would affect their attitudes toward CM.

Applying the RE-AIM framework into our program evaluation based on the original definitions, “Reach” referred to the number or proportion of individuals who received or were affected by the program. “Effectiveness” was defined as the impact of intervention including the delivery of the education content. We referred “Intention to adopt” as the proportion of participants that intended to adopt the suggestions in the program as it was not feasible to measure the actual adoption right after the education program. Similarly, we were not able to evaluate the program using the “Implementation” and “Maintenance” components in a short timeframe.

### Data Analysis

Data were analyzed using SPSS^®^ Statistics Ver. 26.0 (IBM, United States). Descriptive statistics from the evaluation questionnaire are presented as means, standard deviations, and percentage values. In addition, an exploratory analysis was conducted to compare the feedback from caregivers/families vs. other participants (healthcare professionals, members of the public) using Mann–Whitney *U*-test for continuous variables and Fisher's exact test for categorical variables to advice future program planning that may target specific subgroups.

## Results

### Reach

Based on the latest statistics (22, 23), we estimated that there are around 350 active childhood cancer patients and 450 survivors of childhood cancer attending follow-up clinics in public hospitals that can be contacted. We estimated a priori that the program could reach 10% of this targeted population as an initial phase of the pilot program. There were 285 registrants for our two seminars, including 47 children with cancer who had completed treatment or their caregivers, 54 children with cancer who were still undergoing treatment or their caregivers, 112 healthcare professionals, and 72 members of the public. A total of 115 and 59 participants attended the first and second seminars, respectively, and the seminar recording received over 380 views on social media platforms since April 2021. The two seminars reached around 13% of the childhood cancer population, which is slightly over our target reach. The event and related activities were also covered by nine local media agencies (24).

The hardcopy of the educational booklet was distributed to 43 recipients through the clinics and NGO network. In addition, the web-version of the booklet was sent to 67 participants who joined the seminars and downloaded 143 times from November 2021 to January 2022.

### Effectiveness

Of the 174 attendees, 94 (response rate = 54.0%) provided their feedback on the seminars. The results are presented in Table 1. Most respondents found the content of the seminar useful (mean score = 5.04 out of a total score of 6), especially the content on CM advice (dietary and herbal, mean score = 4.99; exercise, mean score = 4.88; acupressure/massage, mean score = 5.27). No significant differences were found between the caregivers of survivors or patients and other participants in terms of their overall perceived usefulness of content and of each topic in the seminars.

**Table 1:** Results of the feedback survey of the seminars.

	**Total** **(*n* = 94)**	**Caregivers** **and families** **(*n* = 18)**	**Other** **participants** **(*n* = 76)**	***P* ^d^**
**Participants**	*n* (%)	*n* (%)	*n* (%)	
Children who had completed treatment	15 (16.0)	15 (16.0)	NA	NA
Children who were still undergoing treatment	3 (3.2)	3 (3.2)	NA	NA
Healthcare professionals	15 (16.0)	NA	15 (16.0)	NA
Members of the public	3 (3.2)	NA	3 (3.2)	NA
**Overall satisfaction with the seminar** ^**a**^	5.24 [0.86]	5.18 [0.87]	5.25 [0.87]	0.76
**Perceived usefulness of overall content** ^**b**^	5.04 [0.90]	5.11 [0.83]	5.03 [0.92]	0.82
**Perceived usefulness of content of each topic** ^**b**^				
(1) Basic CM perspectives on pediatric cancer	4.84 [1.06]	4.78 [1.11]	4.86 [1.05)	0.79
(2) Body constitution characteristics of children	4.61 [1.03]	4.55 [1.21]	4.63 [1.00]	0.86
(3) General CM principles of health promotion, diet and mental	4.84 [0.94)	4.73 [1.01]	4.87 [0.93]	0.64
health in cancer				
(4) CM advice (dietary and herbal)	4.99 [1.00]	4.89 [0.90]	5.01 [1.03]	0.49
(5) CM advice (exercise)	4.88 [1.07)	4.89 [1.13]	4.88 [1.06]	0.96
(6) CM advice (acupressure/massage)	5.27 [0.87]	5.43 [0.79]	5.23 [0.90]	0.66
**Advice that would be adopted** ^**c**^				
Incorporate the recommended food or herbs in their diet	NA	15 (83.3)	NA	NA
Try the traditional Chinese health exercises	NA	10 (55.6)	NA	NA
Take herbal tea	NA	9 (50.0)	NA	NA
Use acupressure/massage	NA	N/A	NA	NA
**Interested in attending future events**	76 (80.9)	18 (100)	58 (76.3)	0.02

*CM, chinese medicine; NA, not applicable*.

*^a^Presented as mean [standard deviation]. Higher score = higher satisfaction. Range: 1–6 points*.

*^b^Presented as mean [standard deviation]. Higher score = higher perceived usefulness. Range: 1–6 points*.

*^c^Only completed by caregivers or families*.

*^d^Mann–Whitney U-test was conducted for continuous variables and Fisher's exact test was conducted for categorical variable*.

With regard to the written educational resources, 18 recipients (response rate = 41.9%) completed the feedback survey (Table 2). Most of them found the overall content useful (mean score = 4.17 out of a total score of 5), especially basic CM perspectives on pediatric cancer treatment and symptom management (mean score = 4.17). Around 90% of the respondents agreed that the education materials raised their interests toward CM (*n* = 17, 94.4%) and they would consider receiving CM treatment in the future during the cancer survivorship phase (*n* = 16, 88.9%).

**Table 2:** Results of the feedback survey of the educational booklet.

	***N* = 18 (%)**
**Overall perceived usefulness of content [Mean (Standard Deviation)] (Total score** **=** **5)**^**a**^	4.17 (1.04)
**Perceived Usefulness of content of each topic [Mean (Standard Deviation)] (Total score** **=** **5)**^**a**^	
(1) Basic CM perspectives on pediatric cancer treatment and	4.17 (1.04)
symptom management	
(2) Body constitution characteristics of children	4.11 (1.02)
(3) General CM principles of diet and CM dietary advice	4.00 (1.19)
(4) Characteristics of CM herbs	4.00 (1.03)
(5) Precautions on using CM herbs	3.94 (1.00)
**Prior use of TCM**	7 (38.9)
**Advice from the booklet that would be adopted**	
Any of the advice	17 (94.4)
Adjust diet based on the CM recommendations	17 (94.4)
Try the recommended CM dietary recipes	17 (94.4)
Use recommended acupressure/massage	17 (94.4)
**Impact of the booklet on attitude toward CM**	
(1) Raise interests toward CM	17 (94.4)
(2) Will consider receiving CM treatment/management in the	16 (88.9)
future	
(3) Will join CM educational events in the future	16 (88.9)
(4) Will recommend other survivors to join CM educational	17 (94.4)
events in the future	

*CM, Chinese medicine*.

*^a^Presented as mean (Standard Deviation). Higher score = higher perceived usefulness*.

### Intention to Adopt

Attendees of the seminars who were caregivers reported that they would help their children to incorporate the recommended foods or herbs in their diet (*n* = 15/18, 83.3%), try the recommended traditional Chinese health exercises (*n* = 10/18, 55.6%), take herbal tea (*n* = 9/18, 50.0%), and perform the recommended acupressure on their children (*n* = 7/7, 100%). Similar results were obtained from the feedback related to the educational materials; nearly all of the respondents (*n* = 17/18, 94.4%) reported that they would adopt the advice provided in the materials.

Besides, some respondents provided free-text responses on their preferences for future seminar topics (*n* = 29). Their suggestions included CM dietary therapy; non-oral therapies, including exercises and acupressure; and precautions, such as herb-drug interactions and foods or herbs unsuitable for cancer survivors. The respondents also recommended the use of illustrations with more concrete case studies to improve their understanding of the material presented. Most of the respondents expressed their interest in attending future events (*n* = 76/94, 80.9%). A higher proportion of caregivers or families of survivors/patients expressed interest as compared to other participants (*P* = 0.02).

## Discussion

This program aims to utilize a hybrid of approaches to educate childhood cancer survivors and their caregivers on the safe and effective use of CM. At the initial stage of implementation, survivors, patients, or their families/caregivers gave positive feedback about the program and they were receptive to the health advice given. Other participants including healthcare professionals were also generally positive toward the education program. Based on these exploratory findings, this may serve as a preliminary framework for incorporating TCIM-related education into the existing risk-based survivorship care. We applied these exploratory findings to further improvise the subsequent components of this educational program:

### Toward Personalization of TCIM Recommendation

The content of our current program is more focused on providing general advice on the safe and appropriate use of CM in survivors of childhood cancer. However, CM is a very personalized treatment; the appropriate CM therapy needs to be tailored according to the physiological response and clinical evaluation of the patient's CM-based body composition. In future, we may consider incorporating a body constitution assessment to facilitate the provision of personalized advice to survivors of childhood cancer during the later phase of the program implementation. Based on the body composition questionnaire results, personalized advice for survivors with different body composition types may also be provided in the educational materials and seminars. This may include recommendations on foods or herbs that may bring more benefits to survivors with specific body composition types, based on CM theories. Apart from program evaluation, future work will include a knowledge assessment of caregivers and older survivors, to evaluate the effectiveness of the program and identify learning gaps that indicate further improvements required to the existing program. This may be particularly important when educating participants on herb-drug interactions and the inappropriate use of herbs or foods that may result in adverse effects on their health. In the future, the education program may also consider other types of TCIM, such as general massage, balneotherapy, and aromatherapy, which may be helpful in rehabilitation among survivors of childhood cancer (9, 25). This can facilitate the collaboration of a multidisciplinary team consisting of oncologists, physiotherapists, psychologists and other healthcare professionals in the integration of TCIM into routine cancer care. Moreover, the needs of survivors of specific cancer types can be considered when developing the educational content. For instance, our team previously found that survivors of CNS tumor were more receptive to non-oral therapies, probably because of their susceptibility to musculoskeletal problems (13); therefore, educational content for these survivors can focus more on non-oral TCIM modalities such as acupuncture and massage.

### Establishing Referral Networks

To enhance the personalized care of survivors, we further propose a referral network that will allow pediatric oncologists, who play a major role in communicating with survivors and their families/caregivers, to refer their patients to complementary medicine practitioners. This referral network would be available to those who would like to seek TCIM advice based on their needs (Figure 1). Complementary medicine practitioners can then collaborate with the oncologists to provide personalized advice to survivors on the use of TCIM, taking into consideration the current health conditions and needs of the survivors. In Hong Kong, the Hospital Authority Tripartite Chinese Medicine Clinics and integrative medical clinics affiliated to academic institutions are examples of institutions that aim to provide TCIM-related patient care services (26). The first flagship Chinese Medicine hospital is also expected to be open in a few years to provide CM and Integrated Chinese-Western Medicine clinical services in Hong Kong (12). The referral network can also be promoted through NGOs to further increase the accessibility of TCIM services. Such an integrated framework can facilitate cooperation among survivors, oncologists, NGOs, and complementary medicine practitioners to optimize survivors' care.

### Promoting Patient Advocacy

Previous studies have shown that tailoring the education resources based on the patients' preferences and their reasons behind using TCIM can facilitate shared-decision making on this topic (27, 28). Our team have previously reported that only a minority of local cancer survivors seek the advice of oncologists before using TCIM (13). The common reasons given by patients or parents for withholding information on TCIM use from clinicians are their belief that TCIM is safe, a fear of the physician's reaction, a lack of knowledge among medical staff, and that the clinician did not ask (29). Therefore, we recognize that patient preferences and advocacy are important when implementing an educational program and establishing a TCIM referral network. Patient advocacy is defined as preserving patients' values and promoting patients' rights to be involved in decision-making. This can lead to patient empowerment (30). To apply this concept to our implementation strategy, we will gather feedback from patients and stakeholders at every phase of the program (Figure 1). For example, in addition to input by registered CM practitioners and pediatric oncologists, all educational resources and proposed topics of the seminars were reviewed by patient advocates and representatives before they are disseminated.

### Continuous Evaluation

Our evaluation attempted to encompass the components of the RE-AIM model (18). As no significant differences were found in the perceived usefulness of content between caregivers of survivors/patients and other participants, future events can continue to target all participants. Future efforts can consider designing more in-depth clinically-oriented content that tailor to the needs of healthcare professionals. At this stage, only some subjective components were assessed, while no objective measures were obtained. Future research will assess other aspects based on the model, especially the long-term impact of the program as the time frame did not allow us to evaluate changes in health practices. The effectiveness of the program can be evaluated based on the behavioral outcomes of the participants, e.g., whether the survivors regularly adopt the CM dietary or exercise recommendations and the subsequent effects on their quality of life, through conducting a follow-up assessment in 6–12 months. Moreover, the maintenance of the intervention effects over time can be determined by periodically collecting data on the effectiveness of the referral service and personalized counseling. We will also continuously evaluate the practical arrangement of the program. For instance, compared with the first seminar, the number of participants who attended the second seminar was lower. While the decrease in attendance was likely due to the progression of the local COVID pandemic situation, responses from the participants, especially healthcare professionals, suggested that they preferred events to be held on weekdays evenings instead of weekend afternoons. Consequently, we might consider such preferences to attract more participants.

### Limitations

Our initial evaluation of the program has some limitations. First, the response rate of the feedback survey was low. This is a common challenge with online surveys. As the community of childhood cancer patients in Hong Kong is small, protecting the identities of the patients is a concern, and hence, we did not collect contact information, other than email addresses, to remind participants to complete the survey. However, additional feedback may be collected during future seminars and other educational events. We expect the response rate to be better when onsite activities are possible after the COVID-19 pandemic subsides. The proposed framework of our educational program may not be generalizable to other settings. For example, our study revealed that the participants generally found the CM advice useful. This high level of receptivity may be due to the recognition of CM by Chinese cancer patients as a self-help process because of a deep cultural grounding in the traditional Chinese philosophy of life in the Chinese society (31). However, the pattern of TCIM use may differ across regions, particularly in Western countries, where CM use has a comparatively lower prevalence (6, 7). Therefore, health authorities should take local preferences into consideration when providing TCIM education for cancer survivors and their caregivers.

## Conclusions

This study provides a framework for structuring a program to educate childhood cancer survivors and their families/caregivers on the safe and effective use of TCIM. With the establishment of the new flagship Chinese Medicine hospital and promotion of quality CM services by the local government in the coming years, such educational programs are important in helping cancer patients achieve better survivorship outcomes through integrative medicine. Overall, we found that participants were receptive to health advice on TCIM. When developing an educational program, local preferences and feedback from participants should be considered. Collaboration with NGOs can also be considered that may increase the accessibility of the education program and materials. Further, a referral network for pediatric oncologists to recommend complementary medicine practitioners should be formed to build an integrative model to optimize the personalized care of survivors.

## Data Availability Statement

The data that support the findings of this study are available from the corresponding authors, YTC and HKK, upon reasonable request.

## Ethics Statement

The studies involving human participants were reviewed and approved by the Survey and Behavioural Research Ethics Committee of the Chinese University of Hong Kong (Reference no. SBRE-20-674). The patients/participants provided their written informed consent to participate in this study.

## Author Contributions

CSL and HWC performed data analysis. CSL and YTC prepared the first draft of the manuscript. All authors contributed to the study conception, study design, and acquisition of data. All authors commented on previous versions of the manuscript, read, and approved the final manuscript.

## Funding

This study was funded by the Chinese Medicine Development Fund (19B1-2/017A_R1), which is a Commissioned Budget by the Food and Health Bureau, the Government of the Hong Kong Special Administrative Region.

## Conflict of Interest

The authors declare that the research was conducted in the absence of any commercial or financial relationships that could be construed as a potential conflictof interest.

## Publisher's Note

All claims expressed in this article are solely those of the authors and do not necessarily represent those of their affiliated organizations, or those of the publisher, the editors and the reviewers. Any product that may be evaluated in this article, or claim that may be made by its manufacturer, is not guaranteed or endorsed by the publisher.
